# Genome Sequence of the Fish Pathogen *Yersinia ruckeri* SC09 Provides Insights into Niche Adaptation and Pathogenic Mechanism

**DOI:** 10.3390/ijms17040557

**Published:** 2016-04-14

**Authors:** Tao Liu, Kai-Yu Wang, Jun Wang, De-Fang Chen, Xiao-Li Huang, Ping Ouyang, Yi Geng, Yang He, Yi Zhou, Jie Min

**Affiliations:** 1 Department of Basic Veterinary, Veterinary Medicine College, Sichuan Agricultural University, 211 Huimin Road, Chengdu 611134, Sichuan, China; liutao1232123@163.com (T.L.); wangjunzl@126.com (J.W.); ouyangping125@163.com (P.O.); gengyisicau@126.com (Y.G.); he_yang_yang@126.com (Y.H.); minjie00awesome@163.com (J.M.); 2 Key Laboratory of Animal Disease and Human Health of Sichuan Province, Sichuan Agricultural University, Chengdu 611134, Sichuan, China; 13981616210@139.com; 3 Department of Aquaculture, College of Animal Science & Technology, Sichuan Agricultural University, Chengdu 611134, Sichuan, China; chendf_sicau@126.com (D.-F.C.); hxldyq@126.com (X.-L.H.)

**Keywords:** *Yersinia ruckeri*, whole-genome sequencing, type III secretion system, niche adaptation, pathogenicity

## Abstract

*Yersinia ruckeri* is the etiologic agent of enteric red mouth disease (ERM), a severe fish disease prevailing in worldwide aquaculture industries. Here we report for the first time the complete genome of *Y. ruckeri* (*Yersinia ruckeri*) SC09, a highly virulent strain isolated from *Ictalurus punctatus* with severe septicemia. SC09 possesses a single chromosome of 3,923,491 base pairs, which contains 3651 predicted protein coding sequences (CDS), 19 rRNA genes, and 79 tRNA genes. Among the CDS, we have identified a Ysa locus containing genes encoding all the components of a type III secretion system (T3SS). Comparative analysis suggest that SC09-Ysa share extensive similarity in sequence, gene content, and gene arrangement with *Salmonella enterica* pathogenicity island 1 (SPI1) and chromosome-encoded T3SS from *Yersinia enterocolitica* biotype 1B. Furthermore, phylogenetic analysis shown that SC09-Ysa and SPI1-T3SS belong on the same branch of the phylogenetic tree. These results suggest that SC09-Ysa and SPI1-T3SS appear to mediate biological function to adapt to specific hosts with a similar niche, and both of them are likely to facilitate the development of an intracellular niche. In addition, our analysis also indicated that a substantial part of the SC09 genome might contribute to adaption in the intestinal microenvironment, including a number of proteins associated with aerobic or anaerobic respiration, signal transduction, and various stress reactions. Genomic analysis of the bacterium offered insights into the pathogenic mechanism associated with intracellular infection and intestinal survivability, which constitutes an important first step in understanding the pathogenesis of *Y. ruckeri*.

## 1. Introduction

*Yersinia ruckeri*, a Gram-negative rod-shaped bacterium, is the etiological agent for enteric red mouth disease (ERM) in various fish species, resulting in severe economic losses in aquaculture [1]. The organism was isolated from salmonid fish with ERM in the United States as early as the 1950s [2]. Subsequently, in the late 1970s to the 1980s, it was introduced to Europe from the USA [3,4,5,6]. Since then, the hosts and geographic distribution of this pathogen has increased. Now, it has been isolated in many countries around the world and spread throughout North and South America, Europe, Australia, South Africa, the Middle East, and China, ranging from different hosts such as rainbow trout, carp, catfish, sturgeon, burbot, and perch [1,2]. Vaccination is the most effective method of combating disease and the first commercially available fish vaccine was an immersion vaccine against ERM [7]. Often vaccination by injection is the most effective means of disease prevention in aquaculture. However, this method is stressful for the fish and labor intensive for the farmer. So, novel oral and immersion delivery methods have captured the attention of researchers and aquarists and some corresponding fish vaccines have been developed [8]. Despite the general administration of a quite effective immersion vaccine in salmonid fish, outbreaks still occur, produced mainly by certain isolates [9,10,11]. These nonmotile isolates were different from previous strains, and they were classified as *Y. ruckeri* biotype 2 [12,13]. Recently, it is one of the most important infectious diseases in *Ictalurus punctatus* aquaculture in China [14].

**Figure 1:**
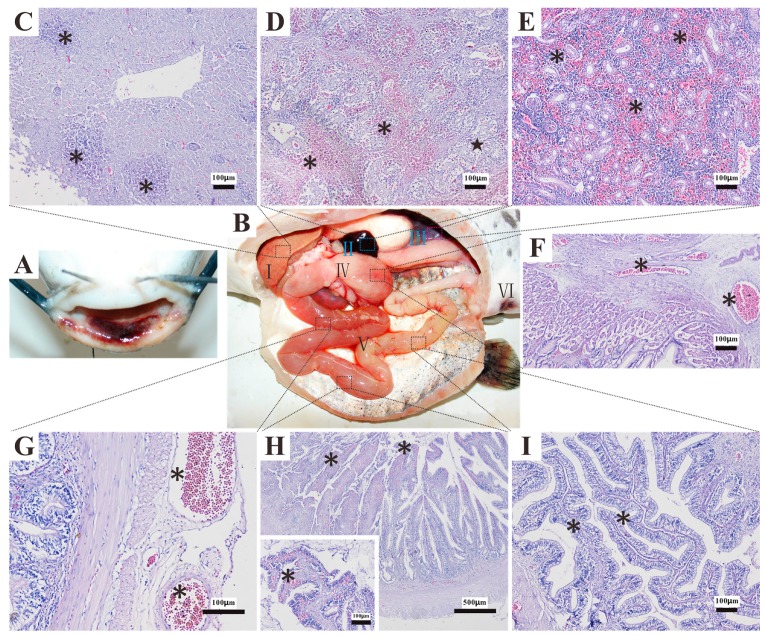
Main symptoms and histopathological changes of the diseased *Ictalurus punctatus*. (**A**) Mandibular hemorrhage in *Ictalurus punctatus* infected with *Y. ruckeri*; (**B**) enlarged liver (**I**), black spleen (**II**), nephremia (**III**) and reddened intestine and anus (IV, V, VI); (**C**) liver coagulation necrosis, necrosis area (*); (**D**) spleen hemorrhage (*) and edema (**★**); (**E**) renal interstitium hemorrhage (*); (**F**) mucosa and serosa of stomach hyperemia (*); (**G**) intestinal serosa hyperemia (*); (**H**) intestinal epithelial cell causing severe necrosis and fall off, intestinal mucosa hemorrhage (*); (**I**) Intestinal villus hyperplasia (*).

ERM caused by *Y. ruckeri* is a serious septicemic disease [1]. Pathological changes in diseased *Ictalurus punctatus* shows general septicemia with inflammation in most organs, including the kidney, spleen, liver, and gastrointestinal tract (Figure 1). The pathogenic mechanisms of *Y. ruckeri* may be associated with its characterization of intracellular infection and intestinal infection. In the process of infection, the organism may invade the gill epithelium and gastrointestinal tract epithelium in the early phase and then enter the blood circulatory system in the later phase, after which it could further infect the spleen and trunk kidney, and accumulate in the lymphoid organs, finally breaking down the immune system [15]. Studies have also shown that *Y. ruckeri* is a facultative intracellular pathogen; it could survive inside macrophages *in vitro* as well as *in vivo* and the number of bacteria inside macrophages steadily increased after immersion infection [16]. The molecular basis of intracellular survival and extracellular intestinal survival on *Y. ruckeri* are still unclear, although both contribute to pathogenicity in fish. In fact, studies into the pathogenic characterization of *Y. ruckeri* are still limited presently, as most researchers’ efforts have focused on individual virulence factors, such as extracellular toxins, high affinity iron uptake system, and resistance to innate immune mechanisms [17,18,19,20,21,22,23,24,25]; systemic research is lacking. In addition, the genetic background of *Y. ruckeri* is still unclear, although some genome sequences of *Y. ruckeri* isolated from salmonid fish have been uploaded onto NCBI [26,27,28]. So, for the purposes of systematic research into *Y. ruckeri*, we utilized second-generation sequencing (SGS) technologies to obtain the complete genome sequence of biotype 1 *Y. ruckeri* SC09 isolated from *Ictalurus punctatus* in Jianyang, China [29]. The genomic analysis provided insights into the niche adaptation of *Y. ruckeri* and countered the blind spots and limitations to our knowledge of the patterns of virulence evolution in *Yersinia*.

## 2. Results and Discussion

### 2.1. Overview of SC09 Genome Sequence

**Figure 2:**
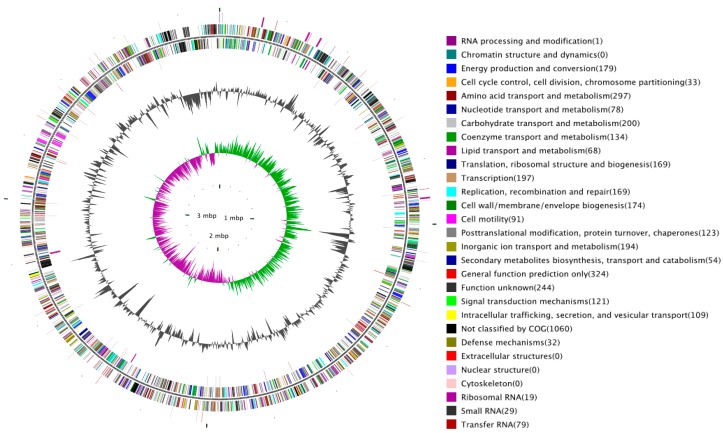
Genome map of *Yersinia ruckeri* SC09. Starting from the outermost ring and moving inwards, the rings show the location of (1) noncoding RNA on the leading strand; (2) all annotated CDS on the leading strand (colors indicating the assigned COG classes); (3) all annotated CDS on the lagging strand (colors indicating the assigned COG classes); (4) noncoding RNA on the leading strand; (5) The two innermost rings show the GC content (black), GC skew+ (green), and GC skew-(dark purple). The legend at the right explains the colors used to indicate the functional COG groups (Figure S4).

We used the Illumina Hiseq2000/Miseq Sequencing platform to yield 3002 Mb paired-end reads (Table S2; Figure S1) that were assembled into 32 contigs (>200 bp) and seven scaffolds (N50 = 3,125,824 bp, Table S3), giving 769-fold coverage of the genome (Figure S2). The seven scaffolds were assembled into a single 3,923,491 bp circular chromosome based on their size (Figure 2). We can clearly observe the GC-skew change at the origin and terminus of replication (oriC), where the leading strand and the lagging strand exchange with each other. However, an obvious region in which GC skew+ changed to GC skew- has occurred at the left side of the oriC, which may be a mark of lateral genetic transfer (LGT) events in the bacterial chromosomes. The general features of the *Y. ruckeri* SC09 genome are summarized in Table 1. This organism contains a 3.9-Mb chromosome with an average GC content of 47.45%, which is the largest genome in all sequenced *Yersinia ruckeri* on NCBI. The coding region accounts for 84.29% of the chromosome and is composed of 3651 coding sequences (CDS), and the gene length most focused on had 100–1400 bp (Table 1; Figure S3). A total of 79 tRNA genes, 19 rRNA genes, representing all 20 amino acids, and 29 sRNA genes were found in the genome. The repetitive DNA sequences in SC09 have been shown in Table 2 and Table 3 (Table S4–S7), which can be used in molecular typing.

### 2.2. Type III Secretion System (T3SS) and Type II Secretion System (T2SS)

**Figure 3:**
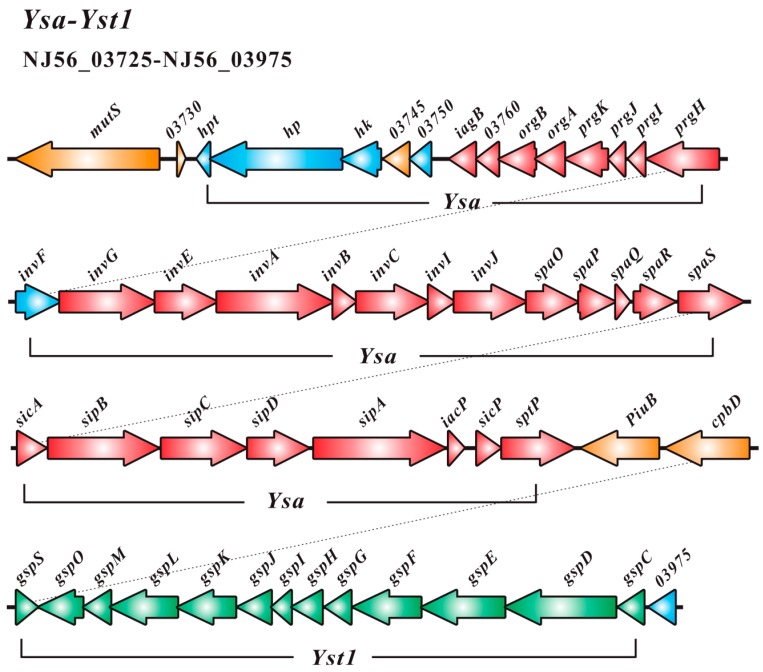
Gene clusters associated with type III secretion system Ysa and type II secretion system Yst1 in SC09.

These days, one of the most interesting findings related to bacterial pathogenesis is the discovery that many pathogens utilize some complex mechanisms to deliver toxins into target eukaryotic cells [30]. These toxins can modulate various cellular functions that are of benefit to the pathogen [31]. The type III secretion system (T3SS) belongs to these protein-delivery machines. T3SSs, as macromolecular nanomachines, are widespread in many Gram-negative bacteria, which involve over 20 different proteins [31]. Among different bacterial species, the overall architecture of T3SSs is similar, but T3SS-encoding operons and the genes encoding for individual components often present a different organizational profile, with the most notable distinctions being identified for genes involved in the regulatory cascade [32]. Often T3SS are found on virulence plasmids, but several systems are dispersed around the chromosome [33]. A T3SS-encoding operon (NJ56_03735- NJ56_03895) was identified from the chromosome in *Y. ruckeri* SC09 and named Ysa (red, Figure 3). The SC09-Ysa is located between 829,763 and 862,347 bps and is located closest to the conserved gene *mutS* (orange, NJ56_03725, encoding DNA mismatch repair protein), associated with the integration of this element into this site, which is similar to SPI1-T3SS (salmonella pathogenicity island 1) [34]. SPI1 is a 40-kb locus encoding a type III secretion system, and acts as a major virulence determinant of *Salmonella enterica* [35]. In the process of infection for *S. enterica*, SPI1 primarily contributes to the invasion of non-phagocytic enterocytes and mediates the inflammatory responses of the intestines [36]. This island also plays a key role in the survival and persistence of bacteria within the systemic compartment of the host [37].

**Figure 4:**
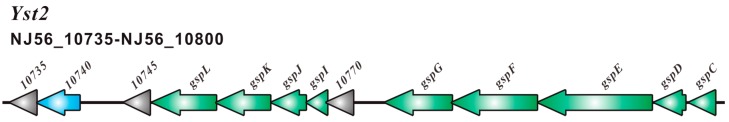
Gene clusters associated with type II secretion system Yst2 in SC09.

The core component of the T3SS is the needle complex, a multi-ring structure that spans the bacterial envelope [38]. Structural organization of the nanomachines is involved in the base, needle, inner membrane export apparatus, cytosolic components, needle tip, and translocon [39]. In SC09-T3SS, the *invG*, *prgH*, and *prgK* (NJ56_03800, NJ56_03790, and NJ56_03775, respectively) encodes the base, which is composed of two rings associated with the inner membrane (encoded by *prgH* and *prgK*) and an outer membrane (encoded by *invG*). Tertiary structure prediction of these three protein-encoding genes indicated that they all share a small domain with an αββαβ configuration (Figure S5); such a domain may be responsible for ring formation [40]. *prgI* (NJ56_03785) and *prgJ* (NJ56_03780) are predicted to encode the needle substructure, assembled from multiple copies of a single residue subunit, and *in silico* modeling has shown that these two proteins share a similar α-helical hairpin shape flanked by flexible regions (Figure S5). Inner membrane export apparatus are encoded by *invA* (NJ56_03810), *spaP* (NJ56_03840), *spaQ* (NJ56_03845), *spaR* (NJ56_03850), and *spaS* (NJ56_03855), which were predicted as channels to transport effectors through the inner membrane [30]. There are several cytosolic proteins that are also essential for the initial secretion of the effectors and they are conserved compared with other components across all T3SSs: an ATPase (InvC), a stalk that links the ATPase to the plasma membrane (InvI), a ring-like component that may act as a sorting platform for ATPase (SpaO), a stator that can recruit the ATPase to the ring-like component (OrgB), and an additional accessory protein (OrgA) [38]; they are respectively encoded by *invC*, *invI*, *spaO*, *orgB*, and *orgA* (NJ56_03820, NJ56_03825, NJ56_03835, NJ56_03765, and NJ56_03770) in SC09-Ysa, of which the ATPase could unfold some effector proteins *in vitro*, and therefore may play an equivalent role *in vivo* [41]. The needle filament is capped at its tip by a single protein [42] and the tip protein is encoded by *sipD* (NJ56_03875) in SC09. The SipD presents a distinct domain organization: an α-helical hairpin located in the N-terminal, a mixed structural elements in the C-terminal region, and a coiled-coil domain located in the central part (Figure S5). This protein is not only responsible for forming the needle tip, but is also thought to provide a platform for the formation of a pore on the host cell membrane [43]. The pore is composed of two interacting translocator proteins [30], SipB and SipC, encoded by *sipB* and *sipC* (NJ56_03865 and NJ56_03870). T3SS-mediated delivery of effector proteins into eukaryotic cells is mediated by the SipB and SipC [30]. Although the translocases are not well conserved at the primary amino acid sequence level (Figure 4), both of them have the α-helical domain with transmembrane helices (Figure S5). In addition, InvB, SicA, SipA, and SicP are type III secretion-associated molecular chaperones for various effector proteins [35] and they are encoded by *invB*, *sicA*, *sipA*, and *sicP*, respectively (NJ56_03815, NJ56_03860, NJ56_03880, and NJ56_03890). InvJ (encoded by *invJ*, NJ56_03830) is responsible for proper needle complex assembly. In the absence of *invJ*, assembly of the inner rod does not take place [44]. An effector protein, SptP (encoded by sptp, NJ56_03895), was identified and contains a tyrosine domain. Once the bacteria are internalized, the SptP can mediate the recovery of the integrity of a host cell membrane by reconstructing the cytoskeleton [45]. Another positive transcriptional activator, InvF (encoded by *invF*, NJ56_03795), belonging to the AraC family (blue, Figure 3), is involved in a regulatory pathway that leads to the activation of the *inv-spa-sic-sip-iac-sic-spt* operon [46]. *hpt*, *hp*, *hk*, and *03750* (NJ56_03735-NJ56_03750) are comprised of a two-component signal transduction system (blue, Figure 3), which may be the central regulator in the overall scheme of SC09-T3SS regulation and activate expression of the *prg/org*, and *inv/spa* operons.

**Figure 5:**
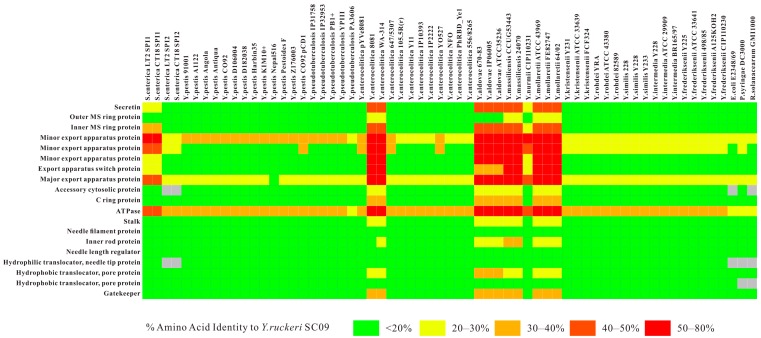
Homologous proteins and their function in various families of T3SSs from different bacteria. Heat map colors are based on the amino acid identity of genes across the T3SS, as indicated in the picture. The percent identities were identified using BLAST searches of the assembled genomes. Amino acid sequences from SC09 act as the comparator sequences.

SC09 also encodes (NJ56_03910–NJ56_03975) a general secretion pathway (GSP)–like system that is known as type II secretion system (T2SS). Genes for all of the core proteins CDEFGHIJKLMOS in SC09-T2SS have been identified (green, Figure 3), denoted as Yst1, which is located closest to Ysa, which is similar to *Y. enterocolitica* 8081 and *Y. enterocolitica* WA-314 (both are in the biotype 1B group) [47]. In addition, *Y. ruckeri* SC09 possesses a second incomplete T2SS (NJ56_10735-NJ56_10800), denoted as Yst2 (Figure 5), which is located outside of the Ysa–Yst1 region. Many Gram-negative bacteria can secrete folded proteins by the type II secretion system (T2SS) [47]. The T2SS actually play an important role in pathogenic or non-pathogenic species [48]. T2SS-Yst1 and T3SS-Ysa are located closest to each other both in *Y. ruckeri* SC09 and highly pathogenic *Yersinia enterocolitica* biotype 1B; this may suggest that Ysa–Yst1 share the same substrates and are likely to facilitate the effects of secretion.

### 2.3. Comparative Analysis of SC09-T3SS and T3SSs from Different Strains

**Figure 6:**
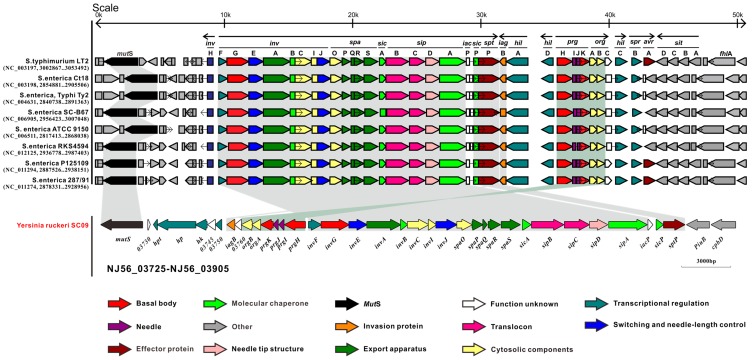
Comparative analysis of the distribution in T3SS between SC09 and SPI1 from *Salmonella*.

**Figure 7:**
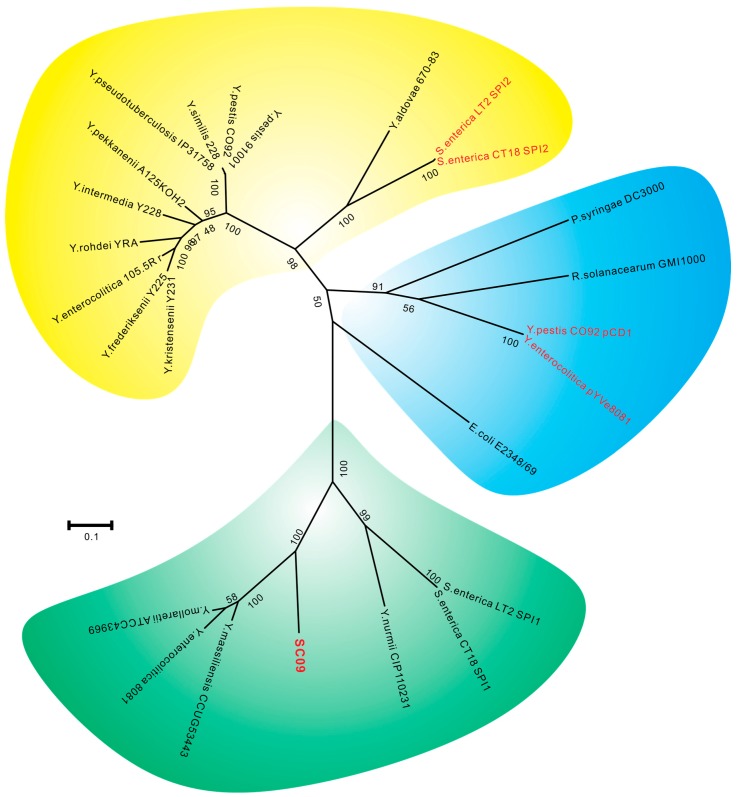
Maximum likelihood phylogenetic tree of the T3SS based on the ATPase amino acid sequences. Values shown on tree branches indicate the percentage of trees among 100 bootstrap replicates carrying that particular branching.

T3SS is a key mechanism for host cell interaction used by a wide range of bacterial pathogens with a variety of lifestyles [32]. Thereby, various types of T3SSs are responsible for various niche adaptations. By comparing the amino acid sequences in homologous proteins of T3SS from different bacteria with the related sequences from *Y. ruckeri* SC09, it is evident that SC09-Ysa is different from the plasmid-encoded T3SSs from *Y. pestis*, *Y. enterocolitica*, and chromosome-encoded SPI2-like T3SSs from *Y. enterocolitica* biotype 1A, 2–5, *Y. pestis*, *Y. pseudotuberculosis*, environmental *Yersinia*, and other types of T3SSs from *E. coli*, *P. syringae*, and *R. solanacearum*; instead, it is closely related to the SPI1-T3SSs and *Y. enterocolitica* biotype 1B encoded T3SSs (Figure 5). Previous studies have found that the Ysa T3SS from *Y. enterocolitica* biotype 1B was closely related to the SPI1-encoded T3SS of *S. enterica* [49]. Further comparative study between SC09-Ysa and SPI1-T3SS from *S. enterica* indicated that the SC09–Ysa share extensive similarity in gene content and gene arrangement with the *Salmonella enterica* pathogenicity island 1, SPI1 (Figure 6). Meanwhile, in consideration of the best-conserved ATPase in T3SSs (due to the fact that it displays high sequence homology among different species [50]), we utilized the amino acid sequences of ATPase to produce a T3SS phylogeny in order to investigate the evolution of SC09-Ysa (Figure 7 and Figure S6). Our phylogenetic study has resolved 25 genera into three species clusters, of which *Y. kristensenii*, *Y. frederiksenii*, *Y. rohdei*, *Y. intermedia*, *Y. pekkanenii*, *Y. similis*, *Y. pestis*, *Y. pseudotuberculosis*, *Y. enterocolitica* (non-biotype-1B), *Y. aldovae*, and *S. enterica* SPI2 form a cluster (yellow, Figure 7); *Y. pestis* plasmid pCD1, *Y. enterocolitica* plasmid pYVe8081, *E. coli*, *P. syringae*, and *R. solanacearum* form the second cluster (blue, Figure 7); and SC09-Ysa, *S. enterica* SPI1, *Y. enterocolitica* biotype 1B and *Y. mollaretii*, *Y. massiliensis*, and *Y. nurmii* form the third cluster (green, Figure 7). Hence, phylogenetic analysis with sequence similarity and gene arrangement clearly suggested that SC09-Ysa, SPI1-T3SS, and chromosome-encoded T3SS from *Y. enterocolitica* biotype 1B may mediate similar biological functions associated with secretion.

All three human pathogenic *Yersinia* species, *Y. pestis*, *Y. pseudotuberculosis*, and *Y. enterocolitica*, carry a pYV virulence plasmid (also named as pCD) that can encode the Ysc type III secretion system and Yops effectors but does not exist in the non-pathogenic population; it was only found in virulent species [51]. Among the three human pathogenic *Yersinia*, *Y. enterocolitica* strains are heterogeneous and can be classified into six biotypes (1A, 1B, 2, 3, 4, and 5) based on biochemical properties [52]. Biotype 1A strains, lacking a high pathogenicity island (HPI) and pYV plasmid, are considered avirulent, whereas biotypes 2–5, only lacking HPI, are low virulence [53]. These five biotypes all belong to the *Y. enterocolitica* subsp. *palearctica* and are usually isolated from Europe and Japan (also known as “Old World” strains) [54]. Most notably, the biotype 1B strains (subsp. *enterocolitica*), carrying both HPI and the pYV plasmid, present the highest virulent among the six biotypes and are mainly found in North America (also termed “New World” strains) [47]. In addition, *Salmonella* isdivided into two species, *bongori* and *enterica*. Both species carry the so-called pathogenicity island 1 (SPI1), a distinct chromosomal operon that encodes a type three secretion system (T3SS-1) [55]. So far, all pathogenic *Salmonella* belong to the *enterica* species and carry an additional pathogenicity island named SPI2, which encodes another type of T3SS (T3SS-2) [55]. These two T3SSs both play a role during the infection of eukaryotes [56]. SPI1 mainly facilitates the invasion of non-phagocytic cells of epithelia [36]. SPI2 is responsible for bacterial survival inside the eukaryotic cell and then lays the foundation for a systemic infection [57]. *Y. ruckeri* SC09 only possesses one SPI1-like T3SS, Ysa, which probably contributes to the development of an intracellular niche in fish. This finding is consistent with the capacity of *Y. ruckeri* to survive in macrophages of rainbow trout [16], and the SC09-Ysa is likely to become the molecular basis for an invasion of the gill epithelium and gastrointestinal tract epithelium.

### 2.4. Metabolism and Transport Systems

**Figure 8:**
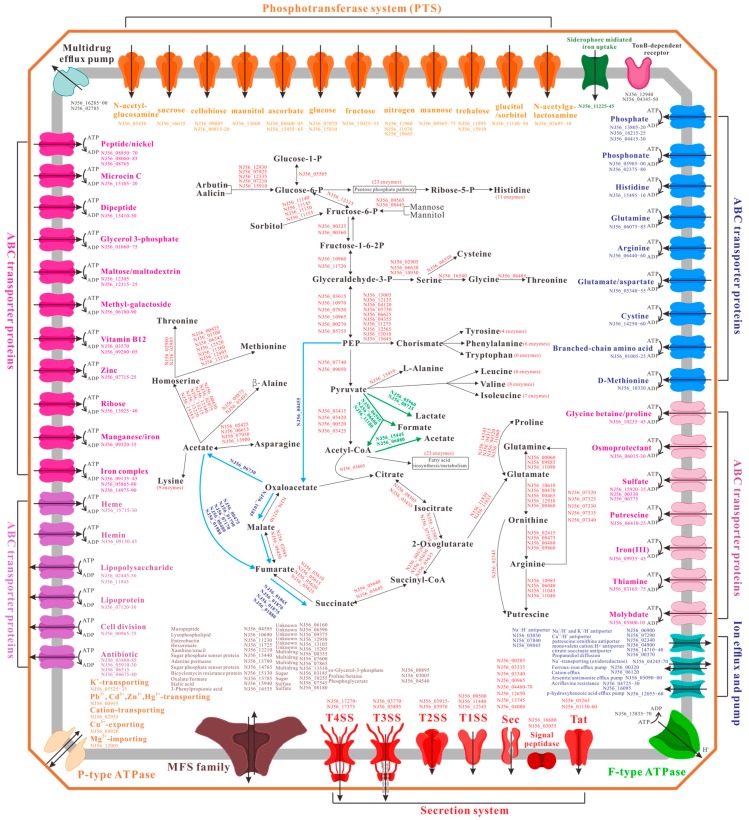
Overview of metabolism and transport in SC09. Different transport families are distinguished by different colors and shapes.

An overview of the basic metabolic pathways of SC09 is presented in Figure 8. Phosphotransferase system (PTS) plays a major role in transport and metabolism of carbon source in SC09. PTS is in fact a multiprotein transport system that can couple the uptake of carbohydrates through the cytoplasmic membrane with their simultaneous phosphorylation [58]. This type of active transport exists exclusively in bacteria [58]. PTS for transport and metabolism of N-acetyl-glucosamine (NJ56_05450), sucrose (NJ56_16635), cellobiose (NJ56_09805, NJ56_09815-20), mannitol (NJ56_13600), ascorbate (NJ56_08600-05, NJ56_15455-65), glucose (NJ56_07070, NJ56_15910), fructose (NJ56_15025-35), nitrogen (NJ56_11960, NJ56_11970, NJ56_10665), mannose (NJ56_09565-75), trehalose (NJ56_11995, NJ56_15910), glucitol/sorbitol (NJ56_11140-50), and *N*-acetylga-lactosamine (NJ56_02695-10) were found in the SC09 genome, all of which allow the bacteria to use carbon sources in a hierarchical manner. The SC09 genome also encodes the complete sets of enzymes necessary for glycolysis, the tricarboxylic cycle (TCA), and the pentose phosphate pathway, while the gluconeogenesis pathway was not identified (lacking the key gene encoding pyruvate carboxylase). Pyruvate is one of the most important fermentation precursors and plays a key role in anaerobic environments [59]. Analysis of the genome of SC09 showed that the pyruvate from glycolysis could be converted into lactate (mediated by NJ56_05960 and NJ56_08725), formate (mediated by NJ56_06595, NJ56_06600 and NJ56_11180), and acetate (mediated by NJ56_15445 and NJ56_06880) for fermentation processes (green arrows, Figure 8). On the other hand, the glycolysis and TCA would change their pathway under anaerobic conditions (blue arrows, Figure 8) in order to adapt to the specific living environments of enteric infection, which is also in agreement with *Y. ruckeri* facultative anaerobic lifestyle in intracellular niches [16]. Additionally, pyruvate, phosphoenolpyruvic acid (PEP), Glyceraldehyde-3-P from glycolysis and Oxaloacetate, and 2-Oxoglutarate from TCA are all precursors in the amino acid synthesis of SC09.

Adding PTS, the SC09 genome contains about 320 genes associated with a wide variety of transport systems, accounting for 8.7% of the total genes, and that is a conservative estimate. Identified transporters are listed in Figure 8, including three types of solute transporting ATPases (P-type, F-type, and ABC-type), major facilitator superfamily (MFS) proteins, ion efflux and pump, siderophore-TonB system, multidrug efflux pump, and various secretion systems (Tat, Sec, T1SS, T2SS, T3SS, T4SS). The P-type ATPases constitute a large protein family that pumps ions and lipids across cellular membranes [60]. Of this type of ATPase, NJ56_05525-NJ56_05535 is predicted to encode a K^+^-transporting ATPase, NJ56_02935 is predicted to encode a cation-transporting ATPase, NJ56_00935 and NJ56_04920 encode P-type ATPases conferring resistance to some toxic metals such as cadmium and copper, and NJ56_12005 is responsible for Mg^2+^-importing. F-type ATPase can use an electrochemical gradient of H^+^ to synthesize ATP [61], which is encoded by NJ56_13835-NJ56_13870 in the genome. Compared to F-type and P-type ATPases, ABC (ATP-binding cassette) transporters are most abundant in SC09. ABC transporters, located in the integral membrane, can actively transport a variety of substrates across the bacterial membranes [62]. In fact, this transporter exhibits specificity to a broad range of substrates in different bacteria: carbohydrates, amino acids, inorganic ions, multidrug, oligopeptides, and osmoprotectants [63]. ABC transporters from SC09 genome enable the bacteria to uptake a variety of amino acids (histidine, glutamine, arginine, glutamate/aspartate, cystine, branched-chain amino acid, d-methionine, glycine betaine/proline,), peptides, vitamin B12, ribose, heme, hemin, thiamine, and inorganic ions, and to exude toxic compounds and antibiotics. Similarly, MFS is also one of the largest groups of transporters in bacteria [64]. MFS transporters in SC09 target a wide spectrum of substrates including enterobactin, hexuronate, xanthine/uracil carbohydrates, lipids, amino acids and peptides, nucleosides, antibiotics, and other unknown molecules. Interestingly, we have identified two types of iron transporters in the SC09 genome; one is homologous to the siderophore-dependent [65] iron acquisition systems (encoded by NJ56_11225-45, NJ56_04345-50, NJ56_11230 and NJ56_12940) that bind ferrichrome as a substrate, and the other belongs to the ABC transporters (encoded by NJ56_09135-45, NJ56_05865-80, NJ56_14975-90, and NJ56_09935-45). So, the uptake of iron may be particularly important for SC09 growth.

The capacity of protein secretion is very important for the niche adaptations and pathogenesis of bacteria [66]. Currently, there are a variety of secretion pathways generally including types 1–8 secretion systems and some additional specific protein transportation systems in different bacteria genomes [66]. Among these systems, the Sec-dependent transport system, twin arginine transport system (Tat), T1SS, T2SS, T3SS, and T4SS were all identified in the genome of *Y. ruckeri* SC09 (Figure 8). We have analyzed T3SS and T2SS, as detailed above. However, unlike other pathogenic *Yersinia* strains or *S. enterica*, *Y. ruckeri* SC09 contains just one copy of SPI1-like T3SS, owing to its specific fish host. 

Secreted proteins from bacteria are normally synthesized with amino terminal signal peptides that target them to either the Sec or the Tat protein export pathway [67]. Protein translocation mediated by Tat (NJ56_01150-60 and NJ56_05265) from SC09 allows for the secretion of fully folded proteins and their chaperones, and appears to be able to mediate various stress responses and virulence. By contrast, the Sec apparatus (NJ56_00285, NJ56_03335, NJ56_03340, NJ56_00965, NJ56_04460-70, NJ56_12650, NJ56_13745, and NJ56_04080) from SC09 appears to translocate polypeptides in an unstructured state. In line with previous findings, SC09 harbors a T4SS (type IV secretion system) [68], encoded by NJ56_17270-NJ56_17375, which is closely related to the *Agrobacterium tumefaciens* VirB system (type IVA). T4SS may take part in the transfer of different effectors into the macrophages or red blood cells, which may facilitate bacterial survival within these cells [69]; this is also in agreement with the facultative anaerobic lifestyle in intracellular niches of *Y. ruckeri*.

### 2.5. Stress Adaptation and Signal Transduction

Bacteria respond to environmental stress generally by some regulatory networks, which can modulate a series of corresponding genes’ expression [70]. These response processes are essential for coping with the complicated stress where temperature, availability of nutrients, and presence of various chemicals are constantly changing [70]. For *Y. ruckeri*, quick adaptation to the dynamic aquatic environment or for survival in fish is generally carried out by a series of stress response systems such as alternative σ factors, various shock proteins, and two-component signal transduction system (TCS), all of which have been identified in *Y. ruckeri* SC09 (Table 4, Table 5 and Table 6).

In bacteria, σ factors are prerequisites for the RNA polymerase [71]. Meanwhile, the σ factors themselves are regulated by anti-σ factors that can bind and inhibit their cognate σ factor, and “appropriators” that deploy a particulars-associated RNA polymerase to a specific promoter class [72]. An array of sigma factors and anti-sigma factors were identified in SC09 (Table 4). Among them, σ-32 and σ^E^, the alternative sigma factors, encoded by *rpoH* and *rpoE* (NJ56_00980 and NJ56_16705), appear to be responsible for the heat-shock response. The heat-shock response is specifically induced by a range of so-called heat-shock proteins (HSPs) as a result of a rapid increase in the environmental temperature (Table 5). In SC09, many of the HSPs are molecular chaperones such as GroEL, GroES, DnaK, DnaJ (encoded by NJ56_01805, NJ56_01800, NJ56_03020 and NJ56_03025), and ATP-dependent proteases (e.g., ClpP, Lon, and HslVU, encoded by NJ56_04615, NJ56_04625, NJ56_00390, and NJ56_00395) that may play a key role in the restoration of protein folding and in protein degradation under normal and stress conditions. The Hsps are essential for protection against various environmental stresses and they can also enhance tolerance to high salt, high temperature, and heavy metals, all of which then contribute to bacterial virulence [73]. These proteins are still highly conserved in nucleotide sequences, whereas the expression and regulation of corresponding genes is highly variable between different organisms and even between various bacteria [73]. In particular, some of the control elements found in SC09 are heat-shock σ factors that regulate the transcription of the major Hsps (Table 4).

On the other hand, it is well known that fish are poikilotherms whose body temperature changes as the ambient water temperature changes. So, the study of cold-shock response is now in the limelight because of the importance of adaptation to cold environments in bacteria [74]. A temperature reduction will result in the inhibition of bacterial growth and proliferation at different levels, and change the effect of protein expression [75]. The synthesis of most cellular proteins is also inhibited after a decrease in temperature [74]. In response to a temperature downshift, members of a family of small cold-shock proteins (CSPs) appear to be induced by SC09 (Table 5). The CSPs tend to interact with single-stranded RNA or DNA and also play a key role in bacteria physiology under both normal and cold situations [76].

In addition, acid shock proteins (encoded by NJ56_08680 and NJ56_10210) may play an important role for *Y. ruckeri* SC09 in facing the gastrointestinal acid environment in fish (Table 5). Furthermore, SC09 also possesses a phage-shock-protein (Psp) system, *pspABCDFG* (encoded by NJ56_08825, NJ56_08820, NJ56_08815, NJ56_08810, NJ56_08830, and NJ56_12255), which appears to correspond to functions that help bacteria manage the impacts of agents impairing cell membrane function (Table 5). The Psp system appears to be widely shared by what has been sequenced so far of the *Enterobacteria* genome (and even across most Gram-negative bacteria genomes), and this system has also been implicated in the virulence of *Salmonella*, *Shigella*, and *Yersinia* [77]. The expression of the *psp* genes is likely driven by RNA polymerase containing the σ-54 factor (encoded by NJ56_11950).

The two-component signal transduction system (TCS), comprised of sensor histidine kinases (HK) and their cognate response regulator (RR) substrates, is one of the most prevalent means by which bacteria sense, respond, and adapt to complex environment changes or their time-varying intracellular state [78]. SC09 harbors a number of TCSs including 23 HK genes and 23 RR genes (Table 6). Among them, NJ56_03735 and NJ56_03740 are predicted to mediate the expression of T3SS in SC09, as mentioned above (Figure 3 and Figure 6). Moreover, the ArcB-ArcA phosphorelay (encoded by NJ56_11820 and NJ56_02970) and CitA-CitB (encoded by NJ56_14745 and NJ56_14750) are associated with the switch from aerobic to anaerobic growth (blue pathway and green pathway in Figure 8) in SC09, which is important for intestinal anaerobic bacteria [79]. The PhoR-PhoB two-component system (encoded by NJ56_04410 and NJ56_04405) allows the organism to sense and respond to changes in phosphate availability. The PhoQ-PhoP pathway (encoded by NJ56_07155 and NJ56_07160) may regulate the expression of a large collection of genes in response to conditions of low Mg^2+^. Meanwhile, chemotaxis in SC09 involves a histidine kinase CheA (encoded by NJ56_14595) that phosphorylates two response regulators, CheY (encoded by NJ56_14565) and CheB (encoded by NJ56_14570). Phosphorylation of each regulator by CheA may be necessary for a proper chemotactic response. The *pgt* system (phosphoglycerate transport system [80]) is probably a TCS (encoded by NJ56_04525, NJ56_04530, NJ56_04535, and NJ56_04540) and may contribute to the development of an intracellular niche for SC09. Other TCSs may also mediate adaptive responses to a broad range of environmental stimuli (Table 6).

## 3. Materials and Methods

### 3.1. Bacterial Growth and DNA Extraction

Biotype 1 *Y. ruckeri* SC09 was isolated from diseased *Ictalurus punctatus* in a reservoir farm in Jianyang, Sichuan province of China and was routinely cultured on a Luria–Bertani (LB) medium at 28 °C. Genomic DNA was isolated from 10 mL overnight culture using the TIANamp Bacteria DNA Kit (TIANGEN Biotech, Beijing, China). DNA was dissolved in TE buffer (10 mM Tris–HCl, 1 mM EDTA, pH 8.0). Genome sequencing was performed by Novogene (Beijing, China).

### 3.2. DNA Sequencing

Two different genomic DNA libraries were constructed according to the manufacturer’s instructions for the Illumina HiSeq 2000 platform (Novogene, Beijing, China) and the Illumina Miseq platform (Novogene, Beijing, China). Long-insert (2–6 kb) libraries were sequenced using Illumina HiSeq 2000 by the paired end mode, and short-insert (500 bp) libraries were sequenced with Illumina Miseq in the manner of paired end mode.

### 3.3. Assembly

Low-quality reads were filtered, and high-quality reads were used for *de novo* assembly. Short reads were assembled using the SOAPdenovo alignment tool (version 2.04), a genome assembler developed particularly for second-generation short-read sequences. The SOAP GapCloser was then used to close gaps where possible after assembly.

### 3.4. Sequence Analysis and Annotation

The protein-coding genes were predicted by the software Glimmer 3.02 [81], while tRNAscan-SE [82] and RNAmmer [83] were used to seek out tRNA and rRNA, respectively. The genome sequence was then uploaded into Rapid Annotation using Subsystem Technology (RAST), which can provide high-quality genome annotations for prokaryotes [84], to find the annotated sequences. The functions of predicted protein-coding genes were subsequently annotated through comparisons with the databases of NCBI-NR, COG, and KEGG. To determine the phylogenetic relationship of T3SS from different genus, we constructed a maximum likelihood phylogenetic tree using MEGA 6.0.6 [85] with the WAG model, further applying 1000 bootstrap replicates. The tree was based on the amino acid sequences encoding T3SS-ATPase. We also used the SWISS-MODEL and TMHMM Server v. 2.0 to predict protein tertiary structure and protein transmembrane regions, respectively, from SC09-Ysa.

### 3.5. Search for Genes and Operons Related to Type III Secretion System (T3SS)

T3SS genes were identified using BLAST searches of the assembled genomes (Table S1). Heatmap colors are based upon amino acid identity across the genes in the operon, as indicated in Figure 5.

### 3.6. Data Availability

The nucleotide sequence of the *Y. ruckeri* SC09 chromosome was submitted to the GenBank database under accession numbers JRWX00000000.

### 3.7. Histopathology

Tissue samples (liver, kidney, spleen, and intestine collected from diseased *Ictalurus punctatus*) were fixed in 10% neutral buffered formalin for one week, and then routinely processed and embedded in paraffin wax. Sections (thickness 0.3 μm) were stained routinely with hematoxylin and eosin, and examined by light microscopy.

## 4. Conclusions

*Yersinia ruckeri* is the causative agent of enteric red mouth disease in various fish species, and this organism is well established as one of the leading fish pathogens hurting the aquaculture industry around the world [1]. The first commercially available fish vaccine was an immersion vaccine against ERM consisting of *Y. ruckeri* bacterin [15]. In this study, we have determined the complete genome sequence of *Y. ruckeri* SC09, a highly virulent strain isolated from *Ictalurus punctatus* that causes severe septicemia. The comprehensive analysis of the genome sequence provides evidence that the SPI1-like T3SS Ysa appears to play an important role in intracellular infection of *Y. ruckeri* and the metabolism pathway associated with anaerobic conditions and the genes encoding TCSs, sigma factors, and shock proteins may be applied to increase SC09 survival ability in intestinal environment of fish. Both of them provide genetic information on niche adaptation and the pathogenic mechanism of *Y. ruckeri*. This study also provides a genetic framework for future studies on the evolution of virulence and physiology characteristics in *Y. ruckeri*.

## Figures and Tables

**Table 1:** General features of the genome of *Y. ruckeri* SC09.

Category	Characteristics
Genome size (bp)	3,923,491
GC content (%)	47.45
Gene number	3651
Gene total length (bp)	3,307,170
Gene average length (bp)	906
Gene length/Genome (%)	84.29
tRNA	79
rRNA	8 × 5 s, 5 × 16 s, 6 × 23 s
sRNA	29

**Table 2:** Information of interspersed repetitive sequences.

Repeat Class	Number	Total Length (bp)	In Genome (%)	Average Length (bp)
LTR	79	6640	0.1692	84
DNA	20	1270	0.0324	64
LINE	24	1786	0.0455	74
SINE	10	711	0.0181	71
RC	1	37	0.0009	37
scRNA	0	0	0	0
Unknown	3	253	0.0064	84
Total	137	10,565	0.2693	78

LTR = long terminal repeat sequence; DNA = DNA transposon; LINE = long interspersed repeated sequence; SINE = short interspersed repeated sequence; RC = rolling circle; scRNA = small cytosolRNA.

**Table 3:** Information of tandem repetitive sequences.

Type	Number	Repeat Size (bp)	Total Length (bp)	In Genome (%)
TRF	212	5~400	23,928	0.6099
Minisatellite DNA	122	11~58	3694	0.0942
Microsatellite DNA	22	5~6	565	0.0144

TRF = tandem repeat sequence.

**Table 4:** Sigma factors and anti-sigma factors in SC09.

Type	CDS	Gene	Annotation
Sigma factor	NJ56_00980	*rpoH*	RNA polymerase factor σ-32
	NJ56_03720	*rpoS*	RNA polymerase factor σ-38
	NJ56_11525	*rpoD*	RNA polymerase σ factor
	NJ56_11950	*rpoN*	RNA polymerase factor σ-54
	NJ56_14270	*fliZ*	Flagellar biosynthesis protein FliZ
	NJ56_14275	*fliA*	Flagellar biosynthesis factor σ-28
	NJ56_16705	*rpoE*	RNA polymerase factor σ-24 (σ^E^)
Anti-sigma factor	NJ56_01355	*rsd*	Anti-RNA polymerase σ 70 factor
	NJ56_04135	*raiA*	σ-54 modulation protein
	NJ56_05160	*rsbV*	Anti-anti-σ regulatory factor
	NJ56_14465	*flgM*	Anti-σ 28 factor
	NJ56_16690	*rseC*	σ-E factor negative regulatory protein RseC
	NJ56_16695	*rseB*	σ-E factor negative regulatory protein RseB
	NJ56_16700	*rseA*	σ-E factor negative regulatory protein RseA
	NJ56_05170	-	Anti-σ regulatory factor (Ser/Thr protein kinase)
	NJ56_11895	-	Anti-σ B factor antagonist

**Table 5:** Shock proteins in *Y. ruckeri* SC09.

Shock Protein	Gene	Feature
NJ56_05240	*csp*	Cold shock protein
NJ56_05245	*cspE*	Cold shock protein
NJ56_06525	*cspD*	Cold shock protein
NJ56_06665	*cspE*	Cold shock protein
NJ56_06670	*cspE/cspD*	Cold shock protein
NJ56_09660	*cspC*	Cold shock protein
NJ56_09775	*cspG*	Cold shock protein
NJ56_09780	*cspG*	Cold shock protein
NJ56_09785	*cspG*	Cold shock protein
NJ56_10190	*cspG*	Cold shock protein
NJ56_14650	*cspC*	Cold shock protein
NJ56_04805	*htpG*	Heat shock protein 90
NJ56_06870	*hspQ*	Heat shock protein HspQ
NJ56_09380	*htpX*	Heat shock protein Htp
NJ56_13685	*ibpB*	Small heat shock protein IbpB
NJ56_13690	*ibpA*	Small heat shock protein IbpA
NJ56_13060	*hslR*	Ribosome-associated heat shock protein Hsp15
NJ56_16745	*grpE*	Heat shock protein GrpE
NJ56_01800	*groES*	Co-chaperonin GroES (HSP10)
NJ56_01805	*groEL*	Chaperonin GroEL (HSP60 family)
NJ56_08730	*hslJ*	Heat shock protein HslJ
NJ56_16480	*hchA*	Chaperone protein HscA
NJ56_16250	*yegD*	Hypothetical chaperone protein
NJ56_13065	*hslO*	Molecular chaperone Hsp33
NJ56_03020	*dnaK*	Molecular chaperone DnaK
NJ56_03025	*dnaJ*	Molecular chaperone DnaJ
NJ56_03140	*djlA*	DnaJ like chaperone protein
NJ56_08565	-	Heat shock protein DnaJ-like protein DjlA
NJ56_00390	*hslU*	ATP-dependent protease ATPase subunit HslU
NJ56_00395	*hslV*	ATP-dependent protease ATPase subunit HslV
NJ56_04615	*clpP*	ATP-dependent Clp protease proteolytic subunit ClpP
NJ56_04620	*clpX*	ATP-dependent Clp protease ATP-binding subunit clpX
NJ56_04625	*lon*	ATP-dependent protease La
NJ56_02295	*deaD*	ATP-dependent RNA helicase DeaD
NJ56_08680	-	Acid shock protein
NJ56_10210	-	Acid shock protein 2 precursor
NJ56_08825	*pspA*	Phage shock protein A
NJ56_08820	*pspB*	Phage shock protein B
NJ56_08815	*pspC*	Phage shock protein C
NJ56_08810	*pspD*	Phage shock protein D
NJ56_08830	*pspF*	Psp operon transcriptional activator
NJ56_12255	*pspG*	Phage shock protein G

**Table 6:** Two-component signal transduction system in *Y. ruckeri* SC09.

Histidine Protein Kinase (HK)	Response Regulator (RR)	HK Gene	RR Gene	Putative Functions
NJ56_14745	NJ56_14750	*citA*	*citB*	Citrate fermentation
NJ56_00055	NJ56_00050	*glnL*	*glnG*	Nitrogen assimilation
NJ56_03735	NJ56_03740	*hp*	*hk*	Type III secretion system
NJ56_00305	NJ56_00310	*cpxA*	*cpxR*	Cell envelop protein folding, degradation
NJ56_02195	NJ56_02200	*basS*	*basR*	
NJ56_11820	NJ56_02970	*arcB*	*arcA*	Anaerobic respiration
NJ56_03590	NJ56_14200	*barA*	*uvrY*	Carbon storage regulation, regulate swarming and quorum sensing
NJ56_07200		*sdiA*		
NJ56_07235	NJ56_07230	*dcuS*	*dcuR*	Anaerobic fumarate respiratory system
NJ56_04410	NJ56_04405	*phoR*	*phoB*	Phosphate limitation
NJ56_07155	NJ56_07160	*phoQ*	*phoP*	Antimicrobial peptide resistance, virulence
NJ56_04530	NJ56_04525	*pgtB*	*pgtA*	Phosphoglycerate transport
NJ56_04785	NJ56_04780	*tctE*	*tctD*	Tricarboxylates transport
NJ56_05520	NJ56_05515	*kdpD*	*kdpE*	Potassium transport
NJ56_13075	NJ56_13080	*envZ*	*ompR*	Osmosis regulation
NJ56_08655	NJ56_08650	*rstB*	*rstA*	Stress
NJ56_16305	NJ56_16310	*baeS*	*baeR*	Multidrug efflux
NJ56_15220	NJ56_15210	*rcsC*	*rcsD*	Capsular polysaccharide synthesis
NJ56_10345		*rcsF*		
NJ56_16600	NJ56_16590	*qseE*	*qseF*	Attaching and effacing lesions
	NJ56_11950		*rpoN*	
NJ56_13445	NJ56_13450	*uhpB*	*uhpA*	Hexose phosphate transport
NJ56_14760	NJ56_14755	*uhpB*	*uhpA*	
NJ56_14595	NJ56_14570	*cheA*	*cheB*	Bacterial chemotaxis
	NJ56_14565		*cheY*	
NJ56_15775	NJ56_15780	Unclear	Unclear	-

## Supplementary Materials

Supplementary materials can be found at http://www.mdpi.com/1422-0067/17/4/557/s1.
