# Supplementary material for: Genome Sequence of the Fish Pathogen Yersinia ruckeri SC09 Provides Insights into Niche Adaptation and Pathogenic Mechanism
Source: Int J Mol Sci. 2016 Apr 14;17(4):557. doi: 10.3390/ijms17040557 (PMC4849013; doi:10.3390/ijms17040557)
Supplement: Supplementary file 1 [file ijms-17-00557-s001.zip › ijms-117898-Supplementary Materials/ijms-117898-supplementary.pdf]

Supplementary Materials Genome Sequence of the Fish Pathogen *Yersinia ruckeri* SC09 Provides Insights into the Niche Adaptation and Pathogenic Mechanism

Tao Liu, Kai-Yu Wang, Jun Wang, De-Fang Chen, Xiao-Li Huang, Ping Ouyang, Yi Geng, Yang He, Yi Zhou and Jie Min

Table S1. Homologous proteins and their function in various families of T3SSs from different bacteria. Heatmap colors are based upon amino acid identity of genes across the T3SS, as indicated. The percent identities were identified using BLAST searches of the assembled genomes. Amino acid sequences from SC09 act as the comparator sequences.
Table S2. Information of sequencing data.
Table S3. Information of scaffolds, contigs and gaps.
Table S4. Information of interspersed repeated sequences.
Table S5. Information of tandem repeat sequences.
Table S6. Information of minisatellite DNA.
Table S7. Information of microsatellite DNA.

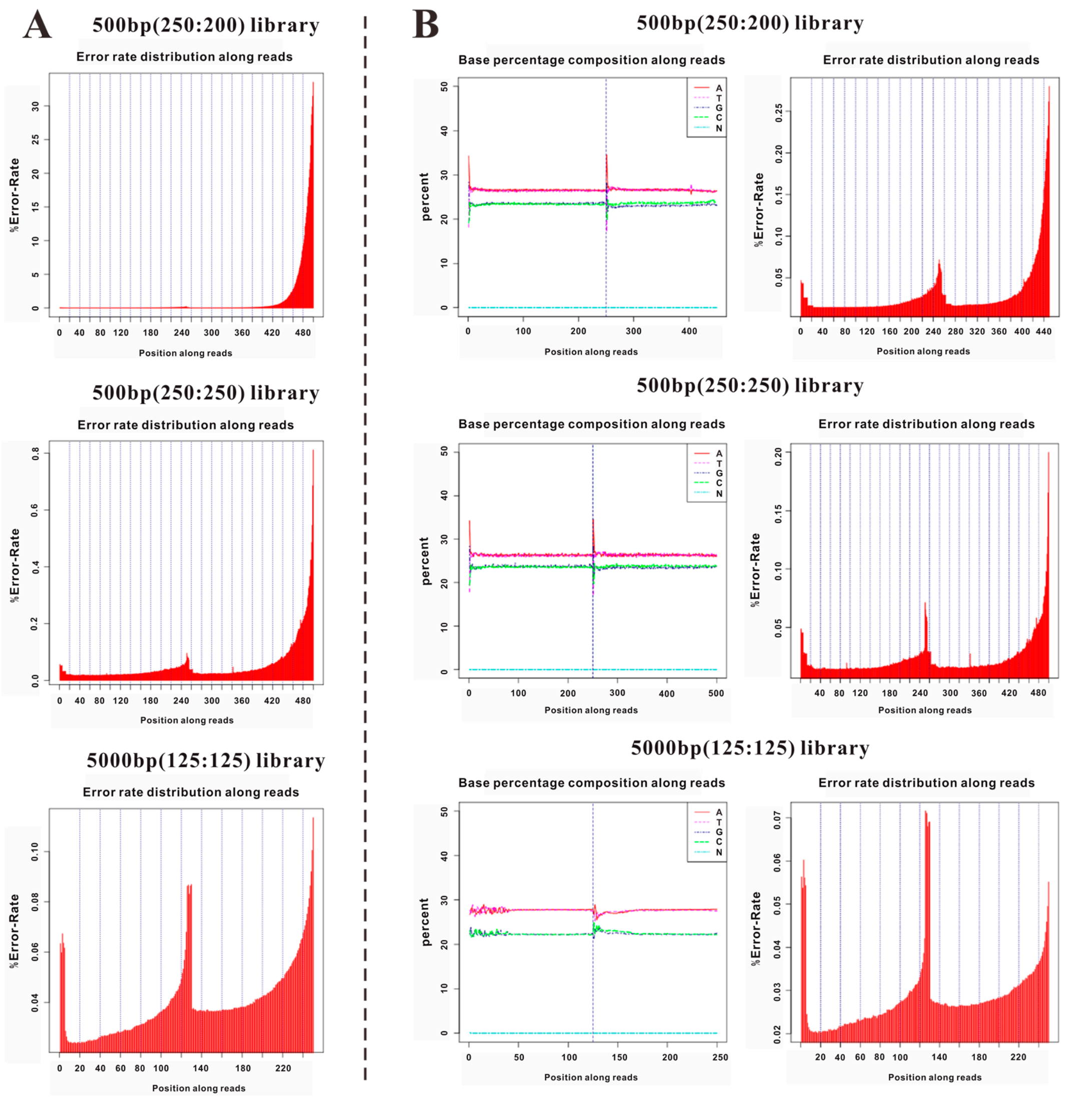

Figure S1. Sequence comparison results before and after data processing (**A**) original data; (**B**) Cleandata.

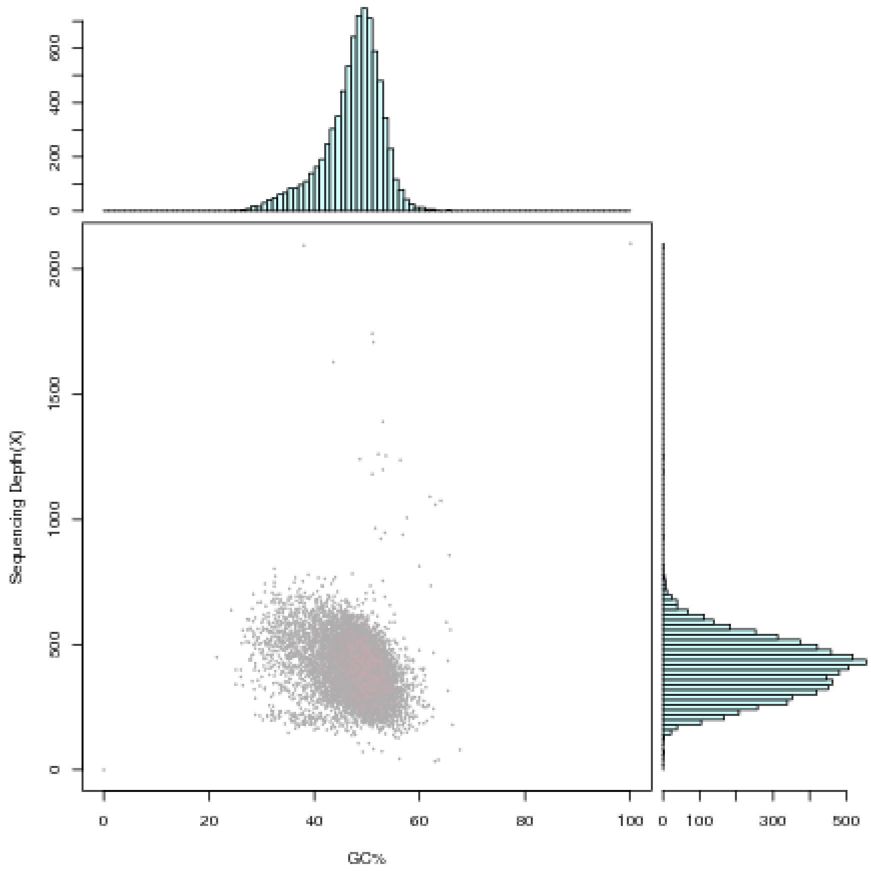

Figure S2. GC-content and Depth analysis chart.

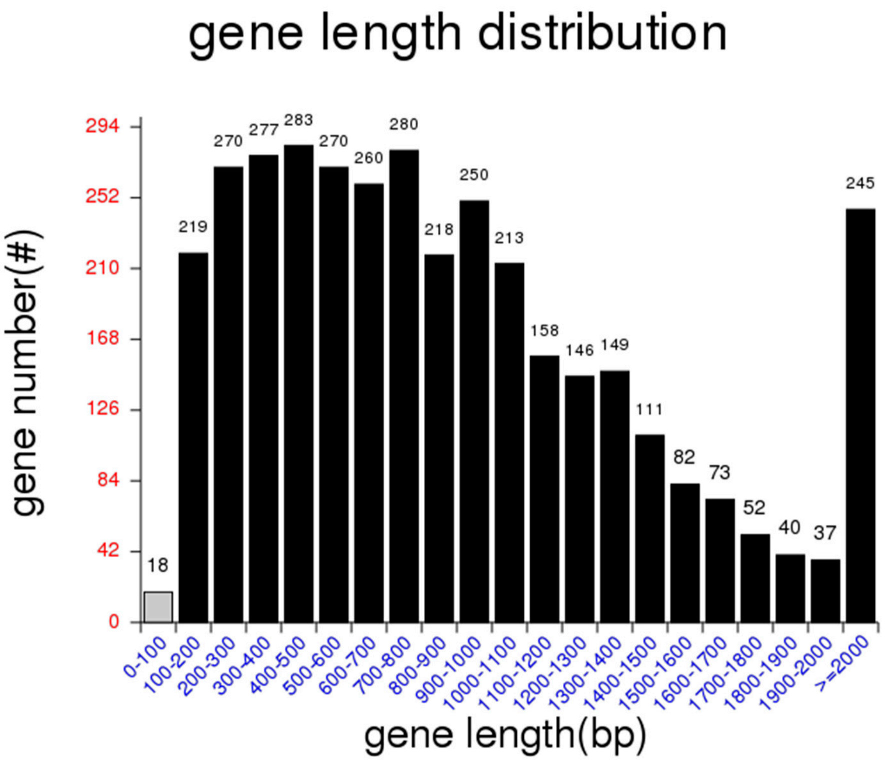

Figure S3. Gene length distribution.

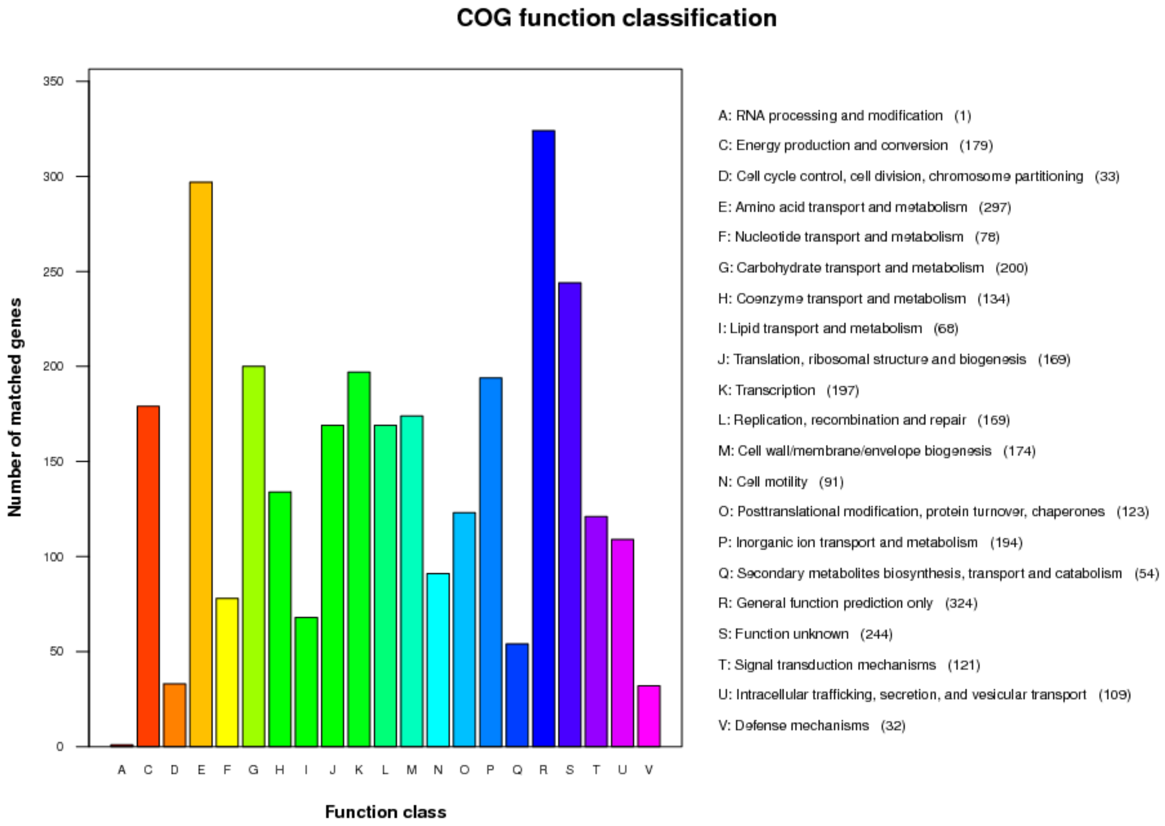

Figure S4. Distribution of proteins based on COG categories.

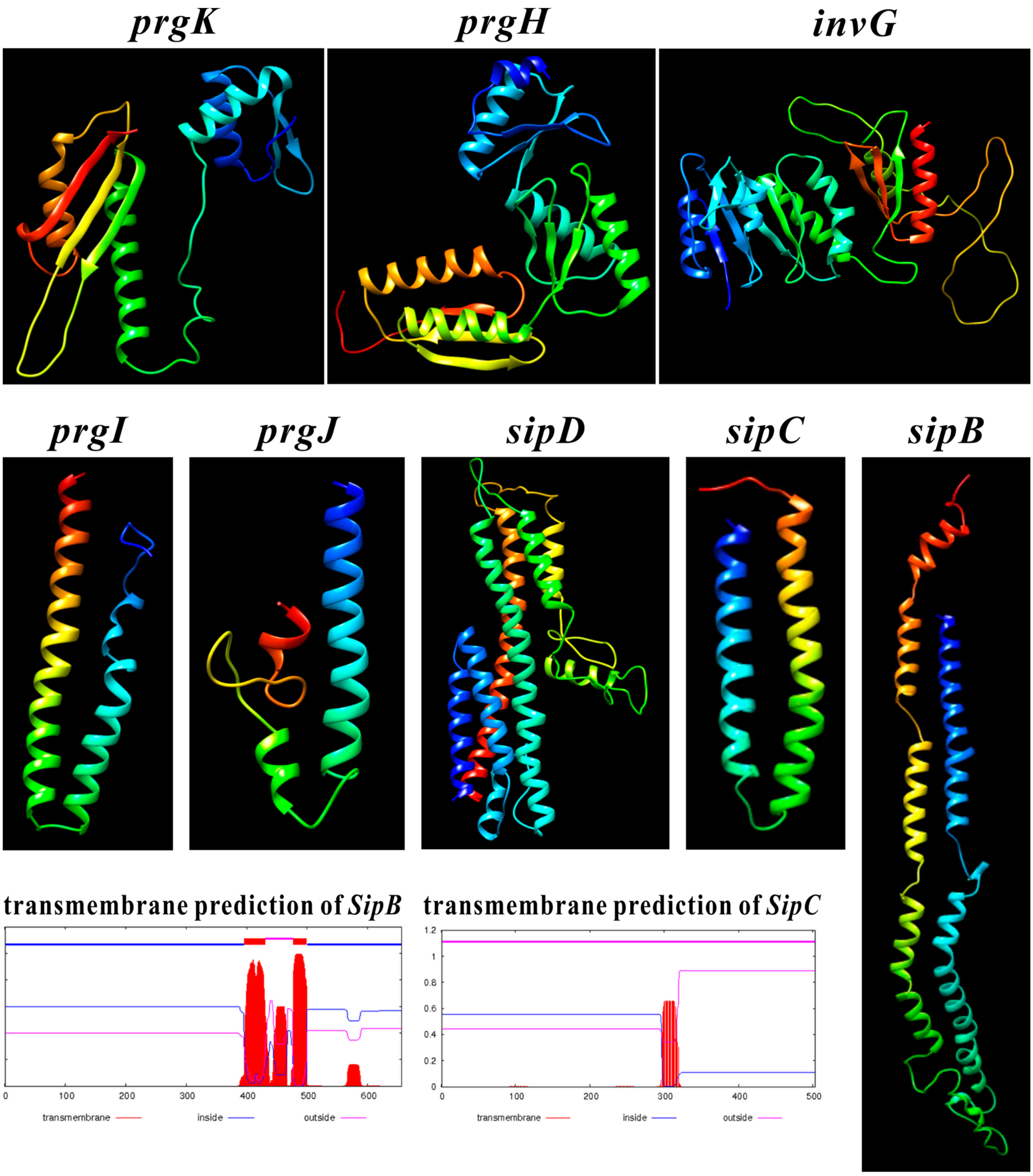

Figure S5. Analysis of protein domain.

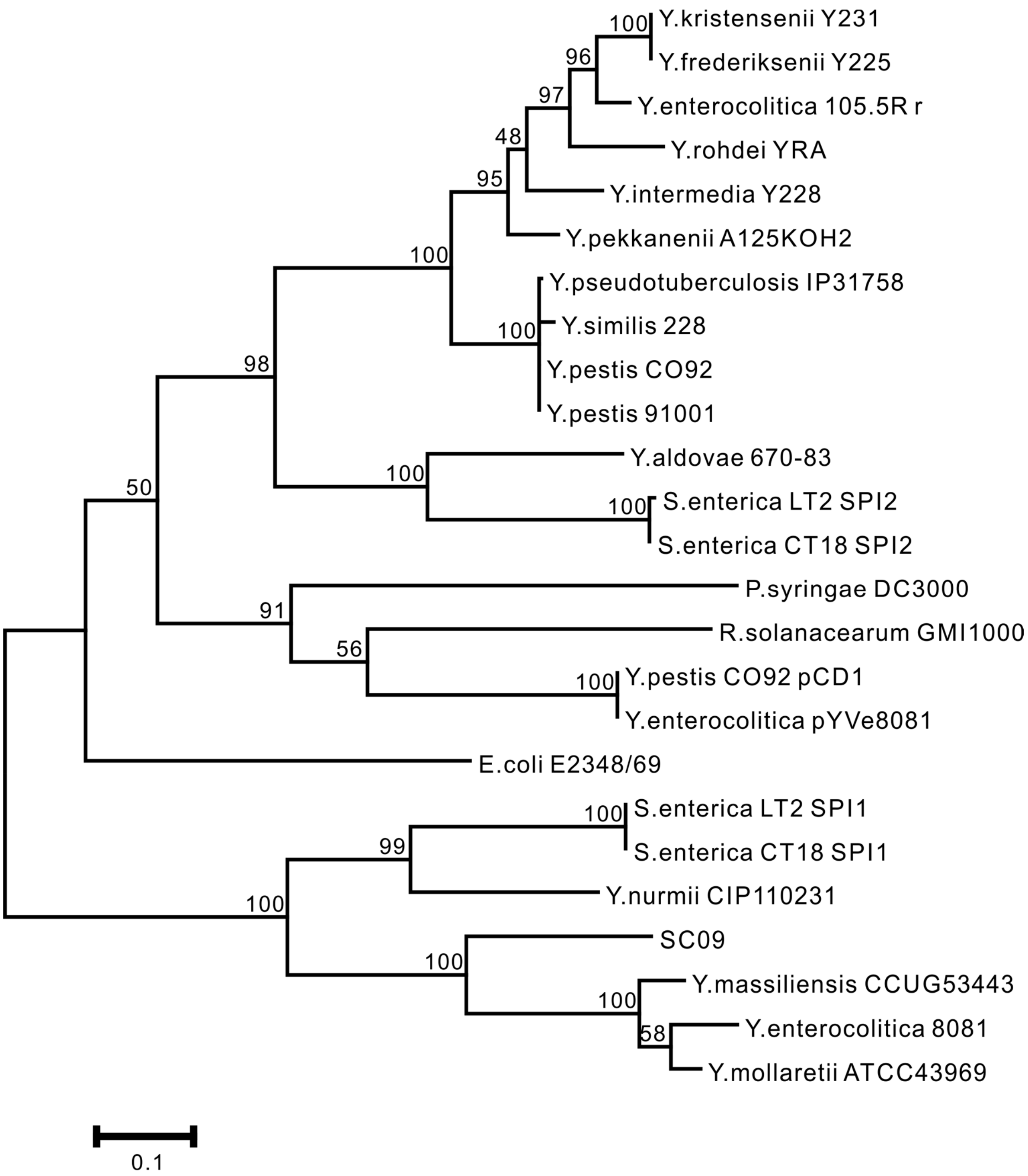

Figure S6. Maximum-likelihood phylogenetic tree (unrooted tree) of the T3SS based on the ATPase amino acid sequences.
